# Dimensional Synthesis for Emulating Nonlinear Disclination Dynamics

**DOI:** 10.1002/nap2.70257

**Published:** 2026-08-26

**Authors:** Junqian Wang, Sihong Lei, Daohong Song, Liqin Tang, Shiqi Xia, Zhigang Chen

**Affiliations:** ^1^ The MOE Key Laboratory of Weak‐Light Nonlinear Photonics TEDA Institute of Applied Physics and School of Physics Nankai University Tianjin China

**Keywords:** 1D synthesized lattices, 3D disclination lattice, higher‐dimensional disclination states, nonlinear dynamics

## Abstract

Nonlinearity plays a central role in topological defect states, governing their stability, localization, and dynamics beyond the linear regime. However, investigating nonlinear dynamics of states in three‐ and higher‐dimensional systems remains highly challenging due to fabrication difficulties and the constraints imposed by spatial dimensionality. Here, we overcome this limitation by employing one‐dimensional synthesized lattices (1DSLs). Using anchor‐site and anchor‐mode mappings, we demonstrate that 1DSLs provide a versatile one‐dimensional platform for emulating the essential nonlinear dynamics and mode‐coupling pathways of disclination states in higher‐dimensional topological lattices. We show that different disclination states exhibit distinct nonlinear dynamics and mode‐coupling pathways, all of which are faithfully reproduced in the mapped 1DSLs. In particular, corner and disclination states exhibit fundamentally different nonlinear coupling mechanisms, whereas three‐dimensional disclination lattices support additional interactions between different classes of defect states. Our work extends dimensional synthesis beyond linear emulation and establishes a practical one‐dimensional platform for investigating nonlinear topological defect physics in higher dimensions. More broadly, it provides a route for exploring nonlinear topological phenomena that are otherwise difficult to access in higher‐dimensional photonic, acoustic, and mechanical wave systems.

## Graphical abstract

(a) Intensity distributions of the 2D disclination lattice under corner‐site excitation in the linear (a1), weakly nonlinear (a2), and strongly nonlinear (a3) regimes. (b) Corresponding one‐dimensional synthesized lattices (1DSLs) obtained through Lanczos mapping under the same nonlinear regimes. As the nonlinearity increases, the system exhibits distinct nonlinear dynamical behaviors, including linear localization (a1, b2), nonlinear diffusion (a2, b2), and nonlinearity‐induced self‐localization (a3, b3). The nonlinear dynamics of the 2D disclination lattice and even higher‐dimensional lattices can be equivalently represented in the mapped 1DSLs. The mapped 1DSLs faithfully reproduce the essential nonlinear dynamics and mode‐coupling pathways, providing a versatile platform for exploring higher‐dimensional nonlinear topological phenomena.

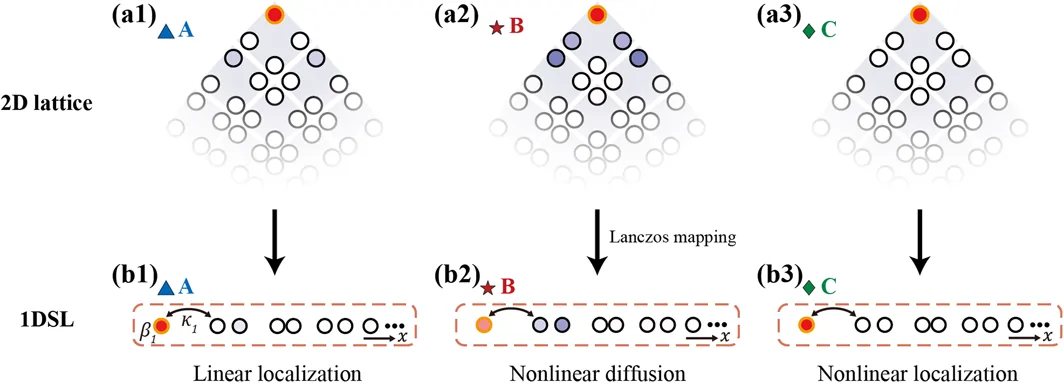

## 1 Introduction

Nonlinearity plays a significant role in topological photonics, giving rise to phenomena such as topological phase transitions [1, 2, 3], nonlinear topological pumping [4, 5, 6], and topological solitons [7, 8, 9]. Nonlinear effects modify topological states through mechanisms including mode coupling and self‐localization, altering their localization, stability, and dynamical properties [10, 11, 12]. In higher‐order topological systems, corner states, owing to their enhanced local intensity, are sensitive to nonlinear effects, which can modify their localization and induce coupling between modes [13, 14, 15, 16]. Beyond boundary and corner states, crystalline defects provide another route to topological localization. Disclination states arise from rotational singularities associated with angular defects in lattice geometry and can host defect‐bound topological states [17, 18, 19, 20]. The physical properties of disclination states in the linear regime have been widely investigated [21, 22, 23, 24, 25]. Beyond the linear regime, nonlinear phenomena in disclination systems have recently attracted increasing attention [26, 27, 28], as nonlinearity can fundamentally alter the stability, localization, and mode‐coupling dynamics of defect states. However, nonlinear disclination dynamics in three and higher dimensions remain largely unexplored and extremely challenging because of fabrication complexity and the constraints imposed by physical spatial dimensionality.

To overcome these difficulties, reduced‐dimensional approaches have emerged as effective strategies for accessing higher‐dimensional physics in experimentally feasible platforms. Among them, synthetic dimensions provide a powerful framework by encoding internal degrees of freedom as additional lattice coordinates, thereby enabling the exploration of higher‐dimensional phenomena in lower‐dimensional structures [29, 30, 31, 32]. This concept has been widely applied in photonic, atomic, and other physical systems to emulate higher‐dimensional lattices and study their associated physical properties [33, 34, 35, 36, 37, 38, 39, 40, 41, 42, 43]. Complementary to such full‐lattice‐emulation approaches, dimensional synthesis offers a fundamentally different strategy. Rather than reconstructing an entire higher‐dimensional system through synthetic dimensions, it selectively maps the dynamics of target states onto a lower‐dimensional platform. In particular, a synthesized lattice is constructed from carefully chosen anchor sites or anchor modes and reproduces the relevant dynamics through properly engineered couplings and on‐site potentials in a one‐dimensional (1D) array [44]. As a result, the dimensionality of the physical platform is substantially reduced, whereas the essential dynamical characteristics of the target states are retained. This distinction makes dimensional synthesis particularly advantageous for studying localized defect states, as it reduces structural complexity while retaining the essential local dynamical features of the targeted defect states. Consequently, it provides an experimentally accessible platform for probing nonlinear defect‐bound states, including disclination states that are otherwise difficult to realize or probe in higher‐dimensional systems.

In this work, we investigate the nonlinear dynamics of higher‐dimensional topological defect states using a one‐dimensional synthesized lattice (1DSL) constructed via Lanczos mapping [45, 46]. Employing this dimensional‐synthesis approach, we show that, although corner and disclination states exhibit similar overall nonlinear localization‐delocalization behavior in a two‐dimensional (2D) lattice, their underlying nonlinear coupling mechanisms are fundamentally different: corner states couple predominantly to edge states, whereas disclination states interact more strongly with bulk states. Extending this analysis to three‐dimensional (3D) structures, we further find that different types of disclination states display distinct nonlinear mode‐coupling pathways, all of which can be faithfully captured within the corresponding 1DSLs. These results demonstrate that dimensional synthesis can capture not only the linear properties of higher‐dimensional defect states but also their nonlinear evolution and mode‐coupling dynamics. Our approach is readily extendable to even higher‐dimensional systems, opening a practical route for exploring nonlinear defect physics in systems that are otherwise inaccessible because of fabrication complexity and dimensional constraints.

## 2 Results

To implement dimensional synthesis, we construct the 1DSL by employing the Lanczos mapping, as detailed in Supporting Information S1: Section S1. This method projects a m‐dimensional (mD, m>1) lattice Hamiltonian $H^{mD}$ onto an effective 1D chain while preserving the linear dynamical evolution initiated from a selected anchor site or mode $|v_{a}\rangle$. The normalized $|v_{a}\rangle$, is chosen as the first Krylov basis vector, that is, the first column of the unitary transformation matrix V and serves as the initial state of the mapped 1DSL. Applying the Lanczos algorithm generates the Krylov basis and yields the effective Hamiltonian $H_{1\text{DSL}}=V^{†}H^{mD}V$, which takes a tridiagonal tight‐binding form

(1) $$H_{1\text{DSL}}=\sum_{n=1}^{N}\beta_{n}|n\rangle \langle n|+\sum_{n=1}^{N-1}\kappa_{n}\left(|n\rangle \langle n+1|+h.c.\right),$$

where $\beta_{n}$ and $\kappa_{n}$ denote the effective on‐site potentials and nearest‐neighbor couplings of the 1DSL, respectively, and N is the total number of sites. Within the dimensional‐synthesis framework, the resulting 1DSL faithfully reproduces the evolution of the selected anchor site or mode while drastically reducing the dimensionality of the physical system. In this way, the 1DSL provides a general route to emulate the dynamics of selected localized states in a reduced 1D lattice independent of the specific dimensionality or geometry of the original lattice.

In the presence of nonlinear excitation, the state evolution is described by the nonlinear Schrödinger equation:

(2) $$i\partial_{t}\psi_{\text{NL}}\left(t\right)=\left[H+\gamma \text{diag}\left({\left|\psi_{\text{NL}}\left(t\right)\right|}^{2}\right)\right]\psi_{\text{NL}}\left(t\right),$$

where $\psi_{\text{NL}}\left(t\right)$ is the site‐amplitude vector in the system with Hamiltonian H, and γ is the nonlinear strength. For convenience, we initialize the mapped 1DSL with $|\psi^{1D}\rangle$ and the original higher‐dimensional mD system with $|\psi^{mD}\rangle$. Throughout the following discussion, the two initial states are normalized, that is, $I^{1D}=I^{mD}=1$, where $I=\sum_{n}{\left|\psi_{n}\right|}^{2}$, enabling a direct comparison between the two systems. The relative nonlinear strength between 1D and mD satisfies $\gamma_{mD}=\mu \gamma_{1D},$ where $\mu =I_{1}^{1D}/\max\left(I_{n}^{mD}\right)$, $I_{n}={\left|\psi_{n}\right|}^{2}$, and $I_{1}^{1D}$ indicates the intensity at the first site (n=1) of the 1DSL. This rescaling of nonlinearity strengths ensures that the maximum‐intensity site in the original mD lattice experiences an effective nonlinear strength equivalent to that in the 1DSL. We adopt this scaling convention throughout the following discussions.

As an example, we consider SSH‐based higher‐order topological disclination lattices hosting corner and disclination states. The basic building block is the 1D SSH chain described by the Hamiltonian

(3) $$H_{\text{SSH}}^{\left(1D\right)}=c_{1}C_{\text{intra}}+c_{2}C_{\text{inter}},$$

$$C_{\text{intra}}=\text{diag}\left(1_{M}⊗\left[\begin{array}{c} 1\\ 0 \end{array}\right],\pm 1\right),C_{\text{inter}}=\text{diag}\left(1_{M}⊗\left[\begin{array}{c} 0\\ 1 \end{array}\right],\pm 1\right),$$

where $c_{1}$ and $c_{2}$ are the intracell and intercell couplings, respectively. By extending the lower‐dimensional SSH dimerization pattern to additional spatial directions, one can construct the higher‐dimensional SSH lattices studied here. The detailed stacking procedure and explicit Hamiltonian forms are provided in Supporting Information S1: Section S2.

We first consider the nonlinear dynamics of a corner state as a representative example in a nontrivial 2D disclination lattice, whose topological properties are inherited from the 2D SSH model. The disclination lattice is generated by removing a sector from the 2D SSH lattice and reconnecting the remaining boundaries (see Supporting Information S1: Section S3 for details) (Figure 1a). The eigenmodes of the 2D disclination lattice can be classified into corner, edge, bulk, and disclination‐center states (Figure 1d1). In the topological regime, the corner state is localized near a single corner. Following the dimensional‐synthesis procedure introduced in Equation (1), the corner eigenmode has dominant weight on the corner site, which is therefore chosen as the anchor site for constructing the corresponding 1DSL and emulating the nonlinear evolution of the associated anchor‐site excitation (Figure 1a). The mapped 1DSL is characterized by effective on‐site potentials $\beta_{n}$ and nearest‐neighbor couplings $\kappa_{n}$, as shown in Figure 1c,d2.

**FIGURE 1:**
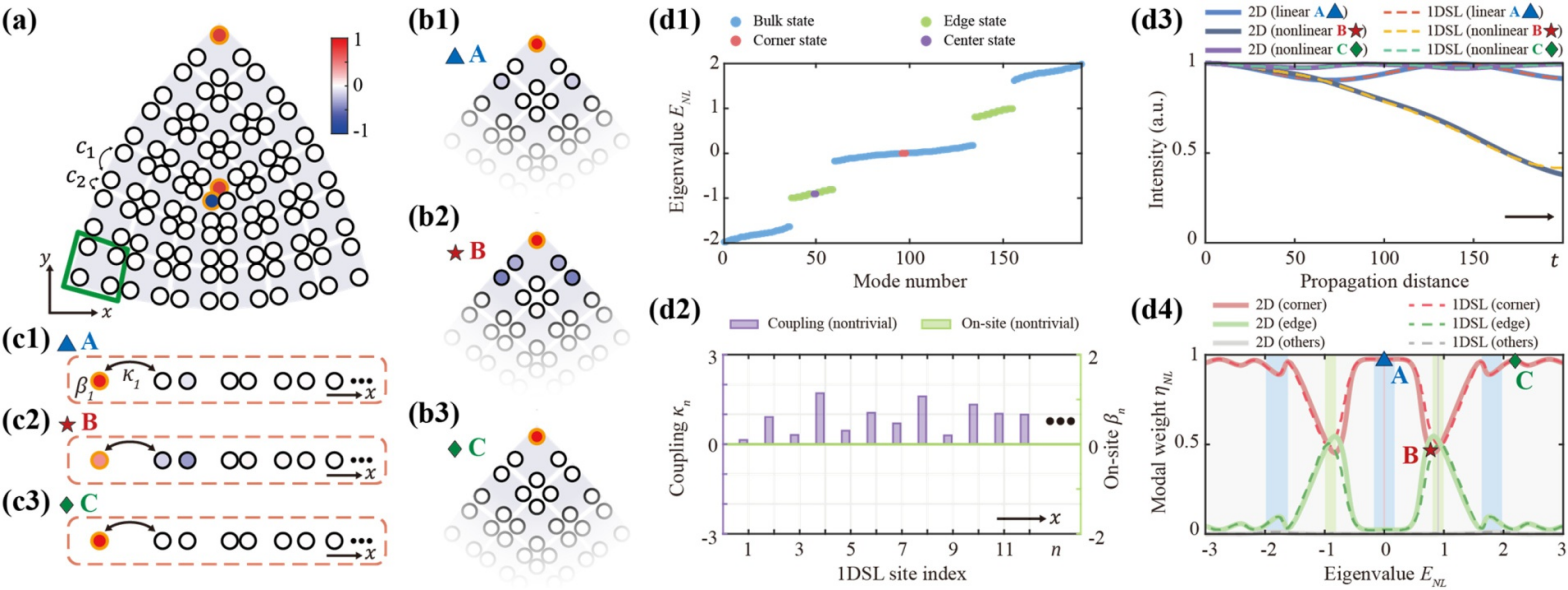
Nonlinear dynamics of a corner state in a 2D disclination lattice and its associated 1D synthesized lattice (1DSL). (a) Schematic of the 2D disclination lattice, showing a corner state at the lattice corner and an out‐of‐phase disclination state at the 2D disclination center. The green square denotes the unit cell inherited from the 2D SSH lattice, and the color bar represents the wavefunction amplitude. The intracell coupling is $c_{1}=0.1$ and the intercell coupling is $c_{2}=0.9$. (b1–b3, c1–c3) Output‐state distributions for different nonlinear strengths, with $\gamma_{2D}=\mu \gamma_{1D}$ and μ=1: (b1, c1) $\gamma_{1D}=0$ (linear regime), (b2, c2) $\gamma_{1D}=1.24$, (b3, c3) $\gamma_{1D}=2.24$. (d1) Energy spectrum of the 2D disclination lattice, where bulk, corner, edge, and disclination‐center states are marked by different colors. (d2) The couplings $\kappa_{n}$ (purple bars) and on‐site potentials $\beta_{n}$ (green bars) of the 1DSL in (c) are obtained via anchor‐site mapping by choosing the red corner site at the top of the lattice in (a) as the anchor site. (d3) Comparison of the evolution of the anchor‐site intensity under different nonlinear strengths in the 2D lattice (b1–b3) and the corresponding 1DSLs (c1–c3). (d4) Relative modal‐weight distribution $\eta_{\text{NL}}$ with respect to the nonlinear energy $E_{\text{NL}}$. The output states in (b, c) and the dynamical evolution in (d3) correspond to the $E_{\text{NL}}$ values indicated in (d4) by the blue triangle, red star, and green diamond, respectively.

In the linear regime, excitation of the first site in the 1DSL reproduces the localized corner‐site dynamics of the original system. In particular, the anchor‐site population in the original lattice is quantified by ${\left|\psi_{1}^{1D}\left(t\right)\right|}^{2}={\left|\langle v_{a}|\psi^{mD}\left(t\right)\rangle \right|}^{2}$, where |ψ(t)〉 denotes the wave evolution governed by Equation (2) with γ=0 in the 1DSL ($\psi^{1D}\left(t\right)$) and the mD lattice ($\psi^{mD}\left(t\right)$). The agreement between the temporal evolutions of these two systems (Figure 1d3) confirms that the 1DSL faithfully reproduces the localized dynamics of the corner state. In the nonlinear regime, as the nonlinearity increases, the effective energy of the initial excitation shifts away from zero energy, thereby modifying its coupling to other states (Figure 1d4). Under weak nonlinearity, both the original system and the mapped 1DSL exhibit similar spreading dynamics, originating from coupling between the initially excited corner state and other eigenmodes of the system (Figure 1b2,c2). At sufficiently strong nonlinearity, the corner‐state excitation becomes strongly localized around the anchor site (Figure 1b3,c3), eventually forming a soliton in the semi‐infinite gap.

To further characterize the nonlinear dynamics, the effective energy of the nonlinear state after a sufficiently long evolution time is defined as $E_{\text{NL}}=\langle \psi_{\text{NL}}\left|H_{\text{NL}}\right|\psi_{\text{NL}}\rangle$ (see Supporting Information S1: Section S4 for details). The nonlinear state mixing is characterized by the overlap $\eta_{\text{NL},\alpha }^{2D}={\left|\langle \psi_{L,\alpha }^{2D}|\psi_{\text{NL}}\rangle \right|}^{2}$, where $|\psi_{L,\alpha }^{2D}\rangle$ denotes an eigenmode of the linear 2D Hamiltonian and α is the eigenmode index. In the 1DSL, the nonlinear dynamics can be directly compared with the mode‐coupling pathways in the original system through $\eta_{\text{NL},\alpha }^{1D}={\left|\langle \psi_{L,\alpha }^{2D}\left|V\right|\psi_{\text{NL}}^{1D}\rangle \right|}^{2}$. The results show consistent behavior in the original lattice and 1DSL: for both focusing and defocusing nonlinearities, as |γ| increases, the initially excited corner state shifts in energy and couples primarily to boundary states, while remaining essentially decoupled from bulk states (Figure 1b2,c2). With a further increase of nonlinearity, this coupling is gradually suppressed and the state evolves into a localized nonlinear soliton (Figure 1b3,c3). These results demonstrate that the 1DSL reproduces not only linear defect‐state dynamics but also nonlinear evolution and mode‐coupling pathways of the topological defect state in the higher‐dimensional system, establishing the consistency of the two descriptions for studying nonlinear dynamics.

Beyond corner states, the 2D disclination lattice also supports topological bound states localized at the disclination center. Unlike corner states, the nonlinear dynamics of disclination‐center states cannot be faithfully captured by anchor‐site mapping (see Supporting Information S1: Section S5 for details), since their wave functions are mainly distributed over two neighboring sites with comparable amplitudes and opposite phases (Figure 1a). To capture this feature, we employ an anchor‐mode mapping, in which the two‐site out‐of‐phase disclination profile is taken as the initial anchor mode. The resulting effective $\beta_{n}$ and $\kappa_{n}$ are shown in Figure 2c1. Under such an anchor‐mode mapping, the evolution of the anchor‐mode amplitude is encoded into the first site of the corresponding 1DSL, allowing the 1DSL to emulate the linear evolution of the disclination state (Figure 2a1,b1). Remarkably, the same mapping remains valid in the nonlinear regime. As the nonlinearity |γ| increases, the effective nonlinear energy $E_{\text{NL}}$ of the disclination‐center‐state excitation shifts away from its initial position, leading to coupling with other states (Figure 2c3). In sharp contrast to the corner state, which mainly couples to edge states, the nonlinear disclination state couples primarily to bulk states, while showing negligible projection onto edge or corner states (Figure 2a2,b2). With further increases in nonlinearity, this energy shift drives the disclination state into the semi‐infinite gap, where it eventually evolves into a localized nonlinear soliton (Figure 2a3,b3). These features are consistently observed in both the original 2D lattice and the mapped 1DSL, demonstrating that the 1DSL can faithfully distinguish and resolve the nonlinear dynamical behaviors of different topological defect states (Figure 2c2,c3). Moreover, although the disclination center in the topologically trivial regime does not support a linear defect mode, sufficiently strong nonlinearity enables a single‐site excitation to form a localized nonlinear soliton in both the original 2D lattice and the corresponding 1DSL (see Supporting Information S1: Section S6 for details). This result highlights the ability of dimensional synthesis to identify and distinguish defect‐state‐specific nonlinear coupling pathways in higher‐dimensional topological lattices.

**FIGURE 2:**
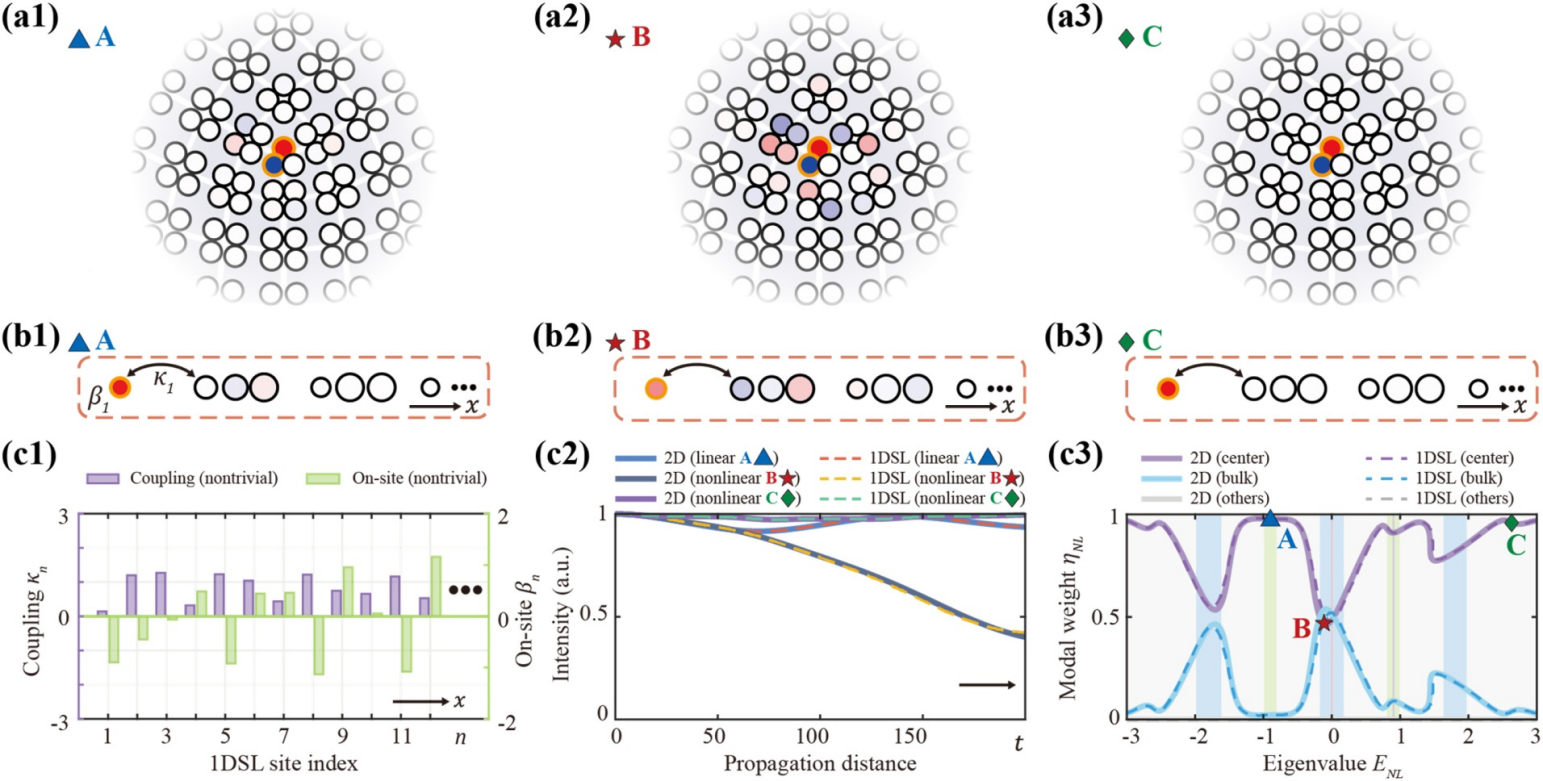
Nonlinear excitation of the disclination‐center state in a 2D disclination lattice and its associated 1DSL. (a, b) Output distributions of the disclination‐center‐state excitation in the 2D disclination lattice (a) and the associated 1DSL (b). The nonlinear strengths in the 1DSL are $\gamma_{1D}=0$ (a1, b1), $\gamma_{1D}=1.24$ (a2, b2), and $\gamma_{1D}=3.44$ (a3, b3). The nonlinear strength in the 2D lattice is related to that in the 1DSL through $\gamma_{2D}=\mu \gamma_{1D}$, with μ=2. (c1) The couplings $\kappa_{n}$ (purple bars) and on‐site potentials $\beta_{n}$ (green bars) of the corresponding 1DSL. (c2) Comparison of the evolution of the projection onto the anchor mode in the 2D lattice (a1–a3) and the 1DSL (b1–b3). (c3) Relative modal‐weight distribution $\eta_{\text{NL}}$ as a function of the effective nonlinear energy $E_{\text{NL}}$. The results in (a, b) and (c2) correspond to the marked $E_{\text{NL}}$ values in (c3). In contrast to the corner state shown in Figure 1, the disclination‐center state exhibits distinct nonlinear mode‐coupling pathways, despite displaying a similar overall localization–delocalization behavior, highlighting the defect‐state‐dependent nature of nonlinear dynamics in disclination lattices.

After verifying the mapping approach in 2D, we construct the 3D SSH lattice (Figure 3a) using the same stacking procedure (see Supporting Information S1: Section S2 for details), which supports corner, edge, surface, and bulk states (Figure 3c1). Here, we excite a single corner site of the 3D lattice and focus on the nonlinear dynamics of the initially excited state during time evolution rather than its propagation along any specific spatial direction of the 3D lattice. Applying the anchor‐site mapping procedure to a corner site of the 3D lattice yields the corresponding 1DSL, whose effective on‐site potentials and nearest‐neighbor couplings are shown in Figure 3c2. The mapped 1DSL reproduces the same linear evolution as the original 3D lattice (Figure 3a1,b1). The nonlinear dynamics of the 3D corner state closely resemble those of its 2D counterpart. With increasing nonlinearity, the input state couples primarily to edge states, rather than bulk states (Figure 3a2,b2), before eventually shifting into the semi‐infinite gap and evolving into a nonlinear soliton (Figure 3c3,c4). The agreement between the original 3D lattice and the mapped 1DSL in both the linear and nonlinear regimes further confirms that the 1DSL provides a reliable reduced‐dimensional description of higher‐dimensional nonlinear corner‐state dynamics.

**FIGURE 3:**
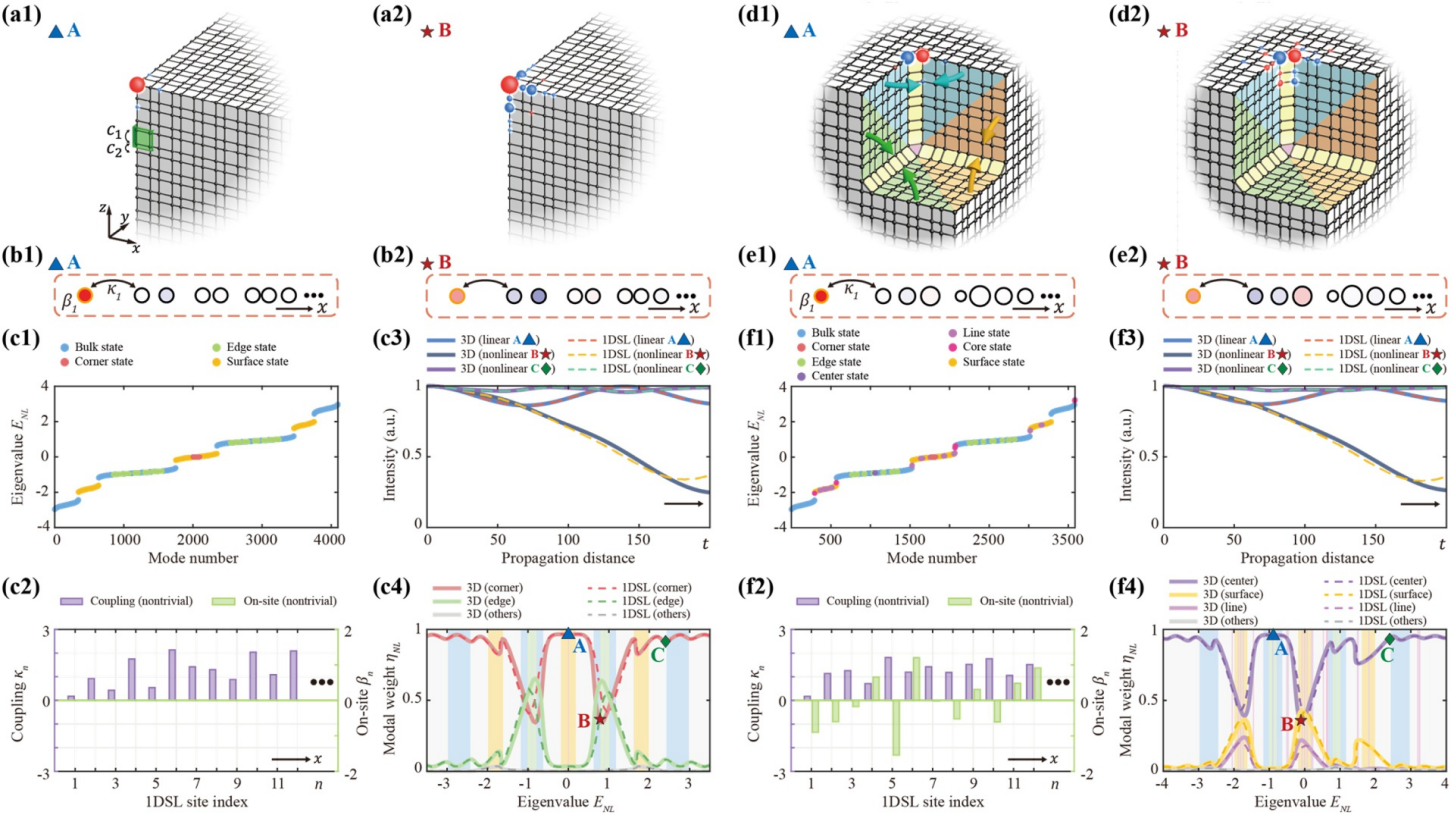
Nonlinear excitation of the corner and disclination‐center states in 3D lattices and their associated 1DSLs. (a1–a2, b1–b2) Output distributions under single‐corner‐site excitation in the 3D SSH lattice and the associated 1DSL in the linear ($\gamma_{1D}=0$) (a1, b1) and nonlinear ($\gamma_{1D}=1.32$) (a2, b2) regimes. The green cube denotes the unit cell, with intracell coupling $c_{1}=0.1$ and intercell coupling $c_{2}=0.9$. The nonlinear strength in the 3D lattice satisfies $\gamma_{3D}=\mu \gamma_{1D}$, with μ=1. (c1) Energy spectrum of the 3D SSH lattice, where different types of states are labeled by different colors. (c2) The couplings $\kappa_{n}$ (purple bars) and on‐site potentials $\beta_{n}$ (green bars) of the associated 1DSL. (c3) Comparison of the nonlinear evolution of the anchor‐corner‐site population in the 3D and the corresponding 1DSL. (c4) Relative modal‐weight distribution $\eta_{\text{NL}}$ as a function of $E_{\text{NL}}$. The results in (a, b) and (c3) correspond to the marked $E_{\text{NL}}$ values in (c4). Panels (d–f) have a layout similar to (a–c), but for the disclination‐center state in the 3D disclination lattice. The nonlinear strength satisfies $\gamma_{3D}=\mu \gamma_{1D}$, with μ=2. Results are shown for the linear regime ($\gamma_{1D}=0$) (d1, e1) and the nonlinear regime ($\gamma_{1D}=1.32$) (d2, e2). The exposed cross‐sectional region in (d1) is partitioned into three colored sections and reconnected along the directions indicated by the thick colored arrows.

We next turn to the nonlinear dynamics of disclination states in the 3D SSH‐based disclination structure. Unlike the 2D case, where a disclination is formed by removing a sector in a plane, the 3D disclination lattice is constructed by excising one‐eighth of a cubic 3D SSH lattice. The exposed cross‐sectional region is then partitioned into three colored sections and reconnected along the directions indicated by the thick colored arrows in Figure 3d1 (see Supporting Information S1: Section S3 for details). This procedure gives rise to a 3D disclination lattice, which supports multiple types of defect states (Figure 3f1), including disclination‐center (Figure 3d), disclination‐line (Figure 4a), and disclination‐core states (Figure 5a). Notably, the latter two states are unique to the 3D geometry and have no counterparts in 2D disclination lattices. Consequently, the 3D system exhibits a richer hierarchy of nonlinear mode‐coupling pathways and defect‐state dynamics than its 2D counterpart.

**FIGURE 4:**
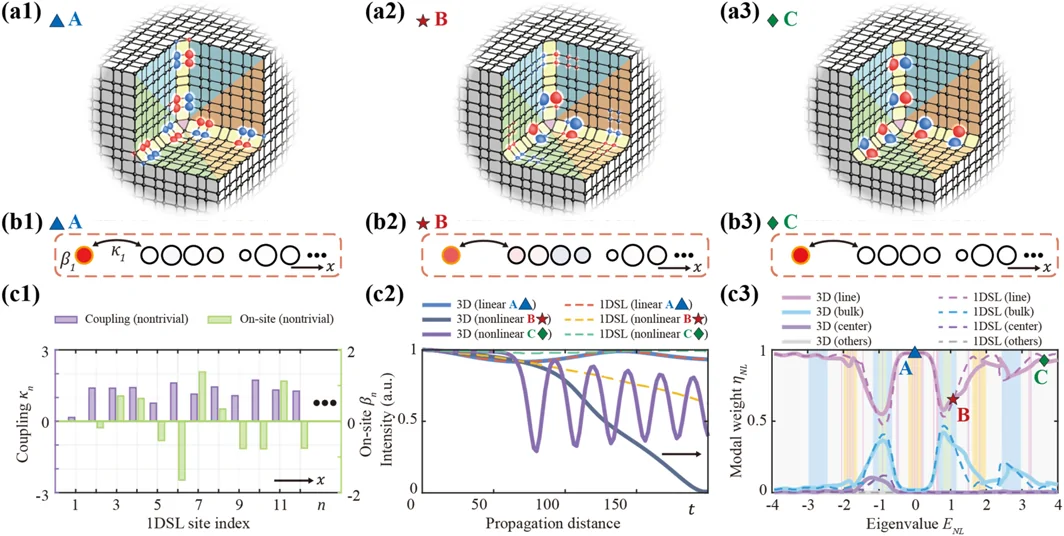
Nonlinear excitation of the disclination‐line state in a 3D lattice and its associated 1DSL. (a1–a3, b1–b3) Output distributions for the excitation of the disclination‐line state in the 3D disclination lattice and the associated 1DSL. The nonlinear strengths in the 1DSL are $\gamma_{1D}=0$ (a1, b1), $\gamma_{1D}=1.36$ (a2, b2), and $\gamma_{1D}=3.52$ (a3, b3). The nonlinear strength in the 3D lattice satisfies $\gamma_{3D}=\mu \gamma_{1D}$ with μ=24. (c1) The couplings $\kappa_{n}$ (purple bars) and on‐site potentials $\beta_{n}$ (green bars) of the associated 1DSL, obtained using the disclination‐line state as the anchor mode. (c2) Comparison of the evolution of the projection onto the disclination‐line anchor mode in the 3D lattice (a1–a3) and the corresponding 1DSL (b1–b3). (c3) Relative modal‐weight distribution $\eta_{\text{NL}}$ as a function of $E_{\text{NL}}$. The results in (a, b) and (c2) correspond to the marked $E_{\text{NL}}$ values in (c3). The disclination‐line state exhibits nonlinear coupling to both disclination‐center and bulk states, demonstrating that the 1DSL captures the dominant nonlinear coupling pathways of this higher‐dimensional defect state.

**FIGURE 5:**
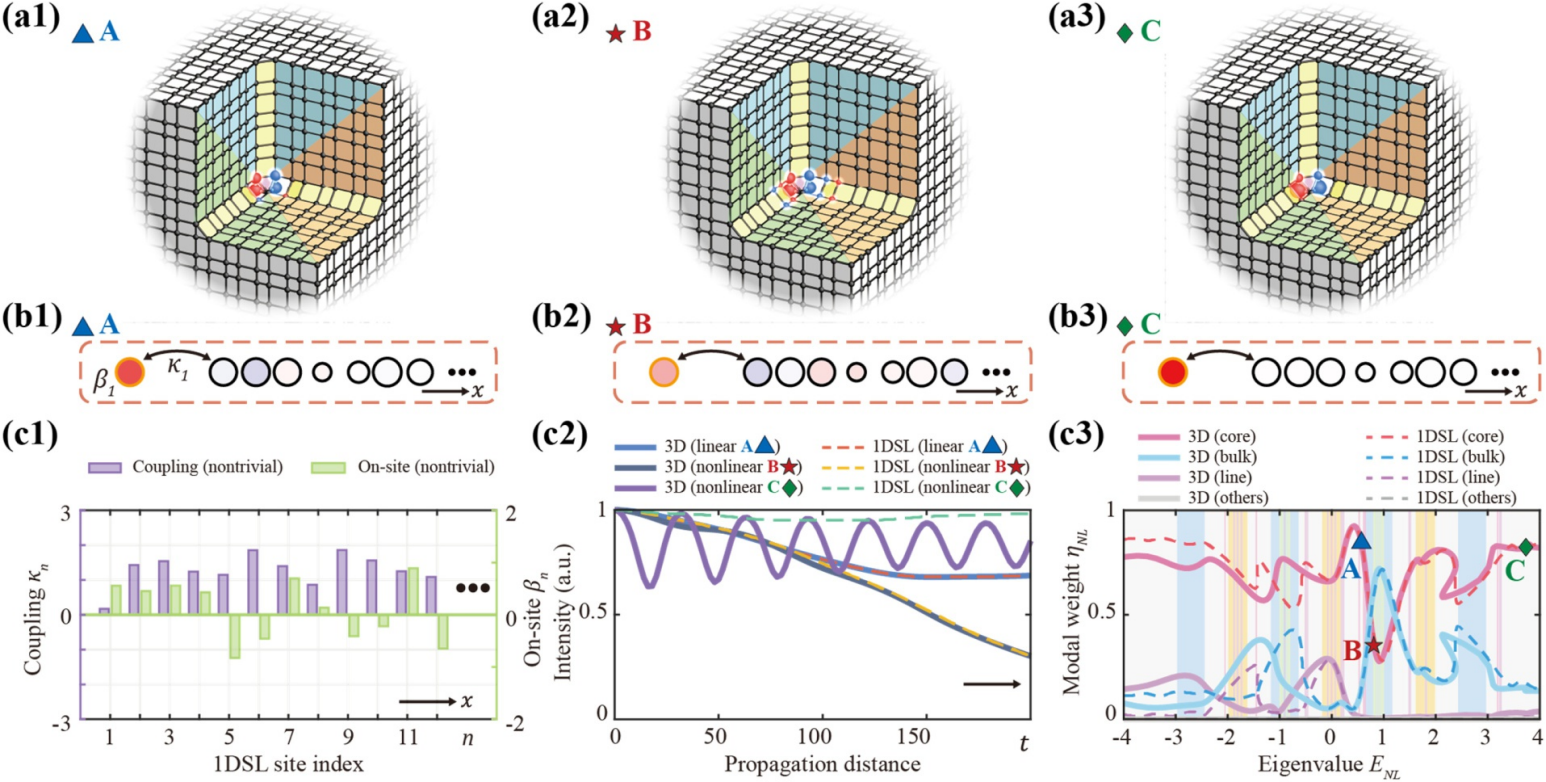
Nonlinear excitation of the disclination‐core state in a 3D disclination lattice and its corresponding 1DSL. The figure has the same layout as Figure 4 but shows the excitation of the disclination‐core state. The nonlinear strengths in the 1DSL are $\gamma_{1D}=0$ (a1, b1), $\gamma_{1D}=0.55$ (a2, b2), and $\gamma_{1D}=2.95$ (a3, b3). The nonlinear strength in the 3D lattice satisfies $\gamma_{3D}=\mu \gamma_{1D}$ with μ=2.76. (c1) Effective nearest‐neighbor couplings $\kappa_{n}$ and on‐site potentials $\beta_{n}$ of the associated 1DSL. (c2) Comparison of the evolution of the projection onto the disclination‐core anchor mode in the 3D lattice (a1–a3) and the corresponding 1DSL (b1–b3). (c3) Relative modal‐weight distribution as a function of $E_{\text{NL}}$. The disclination‐core state exhibits nonlinear coupling to disclination‐line and bulk states, which is faithfully reproduced by the corresponding 1DSL.

To capture the nonlinear dynamics of the 3D disclination‐center state in the reduced 1DSL, we again employ an anchor‐mode mapping (Figure 3f2), since this state, like its 2D counterpart, is not localized on a single site (Figure 3d1). The first site of the mapped 1DSL directly encodes the dynamics of the disclination‐center state (Figure 3e). Both the 1DSL and the original 3D structure show that, under nonlinear excitation, the disclination‐center state couples predominantly to surface states, which are the 3D counterparts of the bulk states involved in the 2D case (Figure 3d2). More importantly, in contrast to the 2D case, the 3D disclination lattice exhibits an additional nonlinear mode‐coupling pathway between different disclination‐induced topological states, which is captured by the 1DSL. In particular, the disclination‐center state can couple to disclination‐line states (Figure 3f4), revealing nonlinear interactions unique to the 3D disclination lattice. As the nonlinearity increases, the effective nonlinear energy $E_{\text{NL}}$ of the disclination‐center‐state excitation shifts, driving the state to interact with these available states before eventually entering the semi‐infinite gap, where a localized nonlinear soliton is formed (Figure 3f3). The same sequence of nonlinear behaviors is observed in the original 3D lattice (Figure 3f3,f4), demonstrating that the mapped 1DSL can faithfully capture the essential nonlinear coupling pathways and dynamical evolution of higher‐dimensional disclination systems.

Besides the disclination‐center state, the 3D disclination lattice also hosts disclination‐line and disclination‐core states, which have no counterparts in the 2D case (Figures 4 and 5). To study their nonlinear dynamics, we again construct the corresponding 1DSLs through anchor‐mode mapping (Figures 4c1 and 5c1). In these cases, however, the nonlinear evolution in the 1DSL and in the original 3D lattice becomes qualitatively different. In the 1DSL, both disclination‐line‐state and disclination‐core‐state excitations can eventually evolve into localized soliton states under strong nonlinearity (Figures 4b3, c2 and 5b3,c2). In contrast, in the original 3D structure, these excitations do not form solitons but instead exhibit competition among different disclination‐related states during evolution (Figures 4a3,c2 and 5a3,c2). This difference originates from the fact that, in the 3D lattice, these defect states spatially overlap with other defect states, causing their nonlinear dynamics to be influenced by inter‐mode competition. By contrast, the anchor‐mode mapping removes the direct real‐space overlap among these states in the 1DSL and isolates the target excitation. As a result, the mapped system emphasizes the intrinsic nonlinear evolution of the selected defect state, whereas the original 3D lattice additionally incorporates nonlinear competition among overlapping defect states. Nevertheless, the 1DSL still captures the essential mode‐coupling pathways between the initially excited defect state and the surrounding mode manifold under different nonlinear strengths (Figures 4c3 and 5c3). These results reveal both the capabilities and the limitations of dimensional synthesis: although nonlinear interactions arising from overlapping defect states are inherently higher‐dimensional, the essential nonlinear mode‐coupling pathways of individual defect states remain accurately captured within the reduced 1D framework.

For disclination‐line‐state excitation, both the 1DSL and the original 3D lattice reveal nonlinear coupling to bulk states and disclination‐center states as the nonlinear strength increases (Figure 4c3). Similarly, for disclination‐core‐state excitation, coupling to disclination‐line states and to bulk states is observed under different nonlinear strengths in both the 3D lattice and the corresponding 1DSL (Figure 5c3). Although the distinct spatial distributions of the mapped and original states can produce some differences in the detailed coupling behavior at particular nonlinear strengths, the essential nonlinear interactions among disclination‐line, disclination‐core, disclination‐center, and bulk states are captured in the 1DSL (Figures 4c3 and 5c3). In particular, the hierarchy of dominant mode‐coupling pathways and their nonlinear evolution are preserved under dimensional synthesis. These results demonstrate that the 1DSL constructed via anchor‐mode mapping provides a reliable framework for investigating nonlinear interactions between defect states and other nearby classes of states. More broadly, they show that dimensional synthesis can faithfully emulate the essential nonlinear mode‐coupling pathways of higher‐dimensional topological defect states even when the full higher‐dimensional dynamics involve additional inter‐mode competition effects.

## 3 Conclusion

In conclusion, we have demonstrated that dimensional synthesis provides an effective framework for emulating and probing the nonlinear dynamics of higher‐dimensional topological defect states using 1DSLs. Through anchor‐site and anchor‐mode mappings, the 1DSLs faithfully reproduce the essential nonlinear evolution and mode‐coupling pathways of corner and disclination states in 2D and 3D SSH‐based disclination lattices. Beyond validating the mapping approach, our results reveal that different classes of topological defect states exhibit fundamentally distinct nonlinear coupling behaviors. Although corner states couple predominantly to boundary states, disclination states interact through bulk‐, surface‐, and defect‐state‐mediated pathways, with 3D disclination lattices supporting additional nonlinear interactions between different classes of disclination states. These findings uncover a rich hierarchy of nonlinear defect‐state coupling pathways that would be difficult to access directly in higher‐dimensional structures.

We further show that dimensional synthesis remains effective in capturing the dominant mode‐coupling pathways even when the full higher‐dimensional nonlinear dynamics involve multiple competing defect states. Since the underlying mapping strategy is readily extendable to four‐dimensional and higher‐dimensional systems, our approach opens a practical route for investigating nonlinear topological defect physics in systems that are otherwise inaccessible because of fabrication complexity and dimensional constraints. More broadly, our work establishes one‐dimensional synthesized lattices as versatile platforms for exploring higher‐dimensional nonlinear phenomena and provides a new route for studying nonlinear interactions among defect, boundary, and bulk states across a variety of wave systems [11, 33, 34, 47], thereby extending dimensional synthesis from a tool for linear emulation to a framework for uncovering nonlinear physics in higher‐dimensional topological systems.

## Author Contributions

**Junqian Wang:** methodology, software, data curation, formal analysis, validation, investigation, visualization, writing – original draft, conceptualization. **Sihong Lei:** supervision, formal analysis, validation, visualization, funding acquisition, writing – original draft, conceptualization. **Daohong Song:** supervision, project administration, writing – review and editing, funding acquisition. **Liqin Tang:** supervision, project administration, writing – review and editing, funding acquisition. **Shiqi Xia:** conceptualization, investigation, validation, formal analysis, supervision, project administration, visualization, funding acquisition, writing – review and editing, methodology. **Zhigang Chen:** writing – review and editing, funding acquisition, validation, project administration, resources, supervision.

## Conflicts of Interest

The authors declare no conflicts of interest.

## Supporting information


Supporting Information S1

## Data Availability

The data that support the findings of this study are available from the corresponding author upon reasonable request.
