# Supplementary material for: Dimensional Synthesis for Emulating Nonlinear Disclination Dynamics
Source: Nanophotonics. 2026 Aug 26;15(17):e70257. doi: 10.1002/nap2.70257 (PMC13672177; doi:10.1002/nap2.70257)
Supplement: Supplementary file 1 [file NAP2-15-e70257-s001.docx]

**Dimensional Synthesis for Emulating Nonlinear Disclination Dynamics**

Junqian Wang,^1,†^ Sihong Lei,^1,†^ Daohong Song,^1^ Liqin Tang,^1^ Shiqi Xia,^1,2^ Zhigang Chen^1,3^

*^1^The MOE Key Laboratory of Weak-Light Nonlinear Photonics, TEDA Institute of Applied Physics and School of Physics,*

*Nankai University, Tianjin 300457, China*

*^2^e-mail:* [*shiqixia@nankai.edu.cn*](mailto:shiqixia@nankai.edu.cn) *^3^e-mail:* [*zgchen@nankai.edu.cn*](mailto:zgchen@nankai.edu.cn)

*^†^These authors contributed equally to this work.*

**Supplementary Material**

**S1: The Lanczos mapping method**

The Lanczos algorithm [1,2] is an iterative method for efficiently computing eigenvalues and eigenvectors, while retaining the spectral properties of the original system and greatly reducing the complexity associated with higher-dimensional matrices. In physics, it is widely applied to large-scale Hamiltonian systems, particularly in condensed-matter physics and many-body quantum systems [3,4]. The Lanczos mapping method can be understood as a unitary transformation that projects a higher-dimensional Hermitian Hamiltonian onto a tridiagonal form [5]. This tridiagonal Hamiltonian admits a direct physical interpretation as a one-dimensional synthesized lattice (1DSL) with nearest-neighbor couplings and on-site potentials [6], while fully retaining the eigenvalue spectrum of the original system.

For an 𝑚-dimensional Hermitian Hamiltonian $H=H^{\dagger}$, the corresponding 1DSL can be constructed via the Lanczos mapping method as follows:

1. **Initialization**
(a) Choose an initial excitation state $\left| \varphi_{\mathrm{seed}} \right\rangle$, and normalize it to obtain the first Krylov basis vector:

$$\left| \varphi_{1} \right\rangle=\left| \varphi_{\mathrm{seed}} \right\rangle/\left\| \left| \varphi_{\mathrm{seed}} \right\rangle\right\|,$$

(S1)

where $\left\| \cdot\right\|$ denotes the Euclidean norm.

(b) Set the initial values $\kappa_{0}=0$ and $\left| \varphi_{0} \right\rangle=0$.
(c) Calculate the first diagonal (on-site) coefficient:

$\beta_{1}=\left\langle\varphi_{1} | H | \varphi_{1} \right\rangle$.

(S2)

2. **Lanczos Iteration**

For $n=1,2,\ldots,m-1$, perform the following steps:

(a) Calculate the unnormalized new basis vector based on three-term recurrence relations:

$$\left| w_{n+1} \right\rangle=H\left| \varphi_{n} \right\rangle-\beta_{n}\left| \varphi_{n} \right\rangle-\kappa_{n-1}\left| \varphi_{n-1} \right\rangle.$$

(S3)

(b) Calculate the sub-diagonal (nearest-neighbor coupling) coefficient:

$$\kappa_{n}=\left\| \left| w_{n+1} \right\rangle\right\|.$$

(S4)

(c) If $\kappa_{n}\neq0$, normalize to obtain the next Krylov basis vector:

$\left| \varphi_{n+1} \right\rangle=\left| w_{n+1} \right\rangle/\kappa_{n}$.

(S5)

Otherwise, the algorithm terminates [7].

(d) Calculate the current diagonal (on-site) coefficient:

$\beta_{n+1}=\left\langle\varphi_{n+1} | H | \varphi_{n+1} \right\rangle$.

(S6)

3. **Construction of the Tridiagonal Hamiltonian**
(a) After $m$ iterations, the unitary transformation matrix is composed by Krylov basis vector, named Krylov basis matrix:

$$V=\left[ \left| \varphi_{1} \right\rangle,\left| \varphi_{2} \right\rangle,\ldots,\left| \varphi_{m} \right\rangle\right].$$

(S7)

(b) In such Krylov basis, the Hamiltonian is represented as a tridiagonal Hamiltonian:

$$H_{1DSL}=\left[ \begin{matrix} \beta_{1} & \kappa_{1} & & & \\ \kappa_{1}^{\dagger} & \beta_{2} & \kappa_{2} & & \\ & \kappa_{2}^{\dagger} & \beta_{3} & \ddots& \\ & & \ddots& \ddots& \kappa_{m-1} \\ & & & \kappa_{m-1}^{\dagger} & \beta_{m} \end{matrix} \right].$$

(S8)

4. **Physical Interpretation**
(a) According to coupled-mode theory, the diagonal elements $\left\{ \beta_{1},\beta_{2},\ldots,\beta_{m} \right\}$ of the tridiagonal Hamiltonian correspond to the on-site potentials of the 1DSL.
(b) The off-diagonal elements $\left\{ \kappa_{1},\kappa_{2},\ldots,\kappa_{m-1} \right\}$ of the tridiagonal Hamiltonian represent the nearest-neighbor couplings between adjacent sites.
(c) At the $n$-th iteration, the unnormalized basis vector denotes $\left| w_{n} \right\rangle$, and the normalized Krylov basis is $\left| \varphi_{n} \right\rangle$.
(d) The original Hamiltonian is Hermitian, satisfying $H=H^{\dagger}$, and the Krylov basis matrix $V$ satisfies $V^{\dagger}V=I$.
(e) The resulting tridiagonal Hamiltonian preserves the eigenvalue spectrum of the original system and provides an exact mapping onto a one-dimensional lattice.
(f) The effective dimension of the 1DSL is determined by the Krylov dimension prior to convergence or termination of the Lanczos iteration.

Based on the Lanczos algorithm described above, an arbitrary Hermitian Hamiltonian can be equivalently mapped onto a 1DSL, with the resulting representation determined by the choice of the initial Krylov basis (i.e., the anchor mode). When the initial excitation is localized on a single lattice site, the 1DSL reproduces the evolution of that site through an anchor-site mapping. More generally, initializing the excitation with a multi-site superposition state leads to an anchor-mode mapping, in which the resulting 1DSL captures the dynamics of the selected state rather than that of a single site. This formulation provides a more flexible description for capturing higher-dimensional dynamics, allowing the evolution governed by the original Hamiltonian to be recast within the effective 1DSL framework [8]. The mapping exactly preserves the corresponding linear dynamics. Although the nonlinear evolution is not guaranteed to be exactly preserved, the 1DSL can still serve as an effective reduced-dimensional platform for probing the essential nonlinear mode-coupling pathways and dynamical behavior of the original system.

**S2: Stacking procedure for higher-dimensional SSH models**

Here, we demonstrate the construction principle of the higher-dimensional SSH lattices used in this work. Starting from a low-dimensional SSH lattice with alternating intracell $c_{1}$ and intercell $c_{2}$ couplings, higher-dimensional SSH models can be constructed by stacking the lower-dimensional lattice along additional orthogonal spatial directions.

Figure S1a shows a one-dimensional (1D) SSH lattice along the $x$ direction. Its unit cell is indicated by a blue rectangle, and the corresponding tight-binding Hamiltonian is given by:

$$H_{\mathrm{SSH}}^{\left( 1D \right)}=c_{1}C_{\mathrm{intra}}+c_{2}C_{\mathrm{inter}},$$

(S9)

$$C_{\mathrm{intra}}=\mathrm{diag}\left( \boldsymbol{1}_{M}\otimes\left[ \begin{aligned} 1 \\ 0 \end{aligned} \right],\pm1 \right), C_{\mathrm{inter}}=\mathrm{diag}\left( \boldsymbol{1}_{M}\otimes\left[ \begin{aligned} 0 \\ 1 \end{aligned} \right],\pm1 \right),$$

where $\boldsymbol{1}_{M}=\left[ 1,1,\ldots,1 \right]^{T}$ is an $M$-dimensional column vector of ones, and $M$ denotes the number of unit cells in the SSH lattice along the $x$ direction. The parameters $c_{1}$ and $c_{2}$ denote the intracell and intercell couplings, respectively.

By stacking the 1D SSH lattice along the $y$ direction with alternating intracell and intercell couplings, a two-dimensional (2D) SSH lattice is obtained, as shown in Fig. S1b. The unit cell of the 2D SSH lattice, marked by a blue square, leads to the Hamiltonian:

$$H_{\mathrm{SSH}}^{\left( 2D \right)}=\boldsymbol{I}_{2M}\bigotimes H_{\mathrm{SSH}}^{\left( 1D \right)}+C_{\mathrm{intra}}\bigotimes\left( c_{1}\boldsymbol{I}_{2M} \right)+C_{\mathrm{inter}}\bigotimes\left( c_{2}\boldsymbol{I}_{2M} \right),$$

(S10)

where $\boldsymbol{I}_{2M}$ is the identity matrix of dimension $2M$, and here $M$ indicates the number of unit cells along the $x$ and $y$ directions.

Further stacking of the 2D SSH lattice along the $z$ direction yields a three-dimensional (3D) SSH lattice (Fig. S1c), where the unit cell is indicated by a blue cube, and the yellow slab represents a 2D layer embedded in the 3D structure (Fig. S1d). The Hamiltonian of the 3D SSH lattice can be constructed from that of the 2D SSH lattice in block Hamiltonian form as:

$$H_{\mathrm{SSH}}^{\left( 3D \right)}=\boldsymbol{I}_{2M}\bigotimes H_{\mathrm{SSH}}^{\left( 2D \right)}+C_{\mathrm{intra}}\bigotimes\left( c_{1}\boldsymbol{I}_{\left( 2M \right)^{2}} \right)+C_{\mathrm{inter}}\bigotimes\left( c_{2}\boldsymbol{I}_{\left( 2M \right)^{2}} \right),$$

(S11)

where $M$ denotes the number of unit cells along the $z$ direction, $\left( 2M \right)^{2}$ is the number of sites in the 2D SSH lattice described by $H_{\mathrm{SSH}}^{\left( 2D \right)}$.

**Fig. S1. Stacking procedure for constructing higher-dimensional SSH lattices.** (a) Schematic of the 1D SSH lattice along the $x$ direction. (b) Construction of the 2D SSH lattice by stacking the 1D SSH lattice along the $y$ direction with alternating intracell and intercell couplings. (c) Construction of the 3D SSH lattice by stacking the 2D SSH lattice along the $z$ direction. (d) Schematic of the resulting 3D SSH lattice with alternating intracell and intercell couplings. The blue rectangle, square, and cube denote the unit cells of the 1D, 2D, and 3D SSH lattices, respectively.

Based on the stacking procedure of higher-dimensional SSH lattices described above, SSH lattices in higher dimensions can be systematically constructed within the tight-binding framework and described by block Hamiltonians. This approach provides a unified framework for higher-dimensional SSH models and yields a systematic block-form representation of their tight-binding Hamiltonians.

**S3: Construction of higher-dimensional disclination lattices**

To construct higher-dimensional disclination lattices, we first consider the simplest case: constructing the 2D disclination lattice from the 2D SSH lattice. As illustrated in Fig. S2a, the 2D SSH lattice is first cut along the red dashed lines, and the shaded region is removed (Fig. S2a1). The remaining cut-edges are then reconnected along the directions indicated by the red arrows (Fig. S2a2). The resulting 2D disclination lattice exhibits $C_{3}$ symmetry, with disclination-center states localized at the lattice center (Fig. S2a3).

This construction can be extended to a 3D disclination lattice with threefold rotational symmetry (Figs. S2b). As shown in Fig. S2b1, a 3D SSH lattice is considered, from which one-eighth of the structure is removed. The exposed cross-sectional region is then divided into three colored sections and reconnected along the directions indicated by the thick colored arrows (Fig. S2b2). This procedure yields a 3D disclination lattice (Fig. S2b3), which hosts disclination-center, disclination-line, and disclination-core states with distinct spatial localizations. Specifically, the disclination-center state (pink) is localized on the lattice surface and exhibits a spatial distribution similar to that in the 2D case (Figs. S2b2 and S2b3). The disclination-line state (magenta) extends from the lattice surface to the geometric core, while the disclination-core state (purple) is confined at the geometric core. Notably, the disclination-line and disclination-core states belong exclusively to the 3D system and do not have 2D counterparts. The coexistence of these distinct defect states provides a richer topological landscape than in the 2D case and forms the basis for the nonlinear mode-coupling pathways investigated in the main text. This construction serves as the basis for studying higher-dimensional disclination states and their associated topological properties.

**Fig. S2. Schematic of higher-dimensional disclination lattices constructed from higher-dimensional SSH lattices.** (a) Construction of the 2D disclination lattice. (a1) The 2D SSH lattice is cut along the red dashed lines and the shaded region is removed. (a2) The cut-edges of the 2D SSH lattice are reconnected along the arrow directions. (a3) Resulting 2D disclination lattice with disclination-center states localized at the lattice center. (b) The construction of the 3D disclination lattice. (b1) One-eighth of the 3D SSH lattice is removed. (b2) The exposed cross-sectional region is partitioned into three colored regions and reconnected along the indicated directions. The pink, magenta, and purple regions denote the disclination-center, disclination-line, and disclination-core states, respectively. (b3) Resulting 3D disclination structure. In (b), the disclination-center, -line, and -core states are localized on the lattice surface, extended from the lattice surface to the geometric core, and localized at the core, respectively.

**S4: Nonlinear strength and energy shift correspondence in the 1DSL mapping**

In this work, the effective nonlinearity in the higher-dimensional lattice does not directly correspond to that in the 1DSL, and depends on the mode distribution of the initially excited state. The nonlinear dynamical evolution of the higher-dimensional lattices and the 1DSLs shows good agreement only when the nonlinear relation $\gamma_{mD}=\mu\gamma_{1D}$ is satisfied, where $\mu={I_{1}^{1D}}/{max(I_{n}^{mD})}$ represents the ratio between the maximum intensities of the peak-normalized states in the 1DSL and the $m$-dimensional lattice. Here $I_{n}=\left| \psi_{n} \right|^{2}$ and $I_{1}^{1D}$ indicates the intensity on the first site ($n=1$) of the 1DSL. This scaling ensures that the site experiencing the strongest nonlinear response in the original higher-dimensional lattice is matched to the effective nonlinear response at the first site of the mapped 1DSL. Consequently, the nonlinear energy shift induced by the excitation remains comparable in the two systems, enabling a meaningful comparison of their nonlinear mode-coupling pathways.

For the corner states in the 2D SSH and disclination lattices, the anchor-site mapping confines the nonlinear dynamics onto a single anchor site, allowing the 1DSLs to capture the nonlinear dynamical evolution of the initial excitation under $\mu=1$. The nonlinear energy shifts are quantified by the modal effective nonlinear energy $E_{NL}=\left\langle\psi_{NL} | H_{NL} | \psi_{NL} \right\rangle$, calculated under varying nonlinear strengths following the relations above (Figs. S3a and S3c), where $\left| \psi_{NL} \right\rangle$ denotes the nonlinear eigenstate. Under both self-focusing and self-defocusing nonlinearities, the nonlinear energy shifts of corner states in the 2D and 3D disclination lattices traverse the entire band spectrum, while the 1DSLs exhibit consistent behavior.

In contrast, the 2D and 3D disclination-center states involve initial excitations distributed over two out-of-phase sites. The nonlinear strengths in the 2D/3D lattices and the 1DSL satisfy $\gamma_{2D}=\mu\gamma_{1D}$ and $\gamma_{3D}=\mu\gamma_{1D}$ with $\mu=2$. These disclination-center states exhibit nonlinear dynamical behavior consistent with the corresponding 1DSLs under both self-focusing and self-defocusing nonlinearities (Figs. S3b and S3d).

For the disclination-line-state excitation in the 3D disclination lattice, the anchor-state mapping involves an excitation composed of 24 lattice sites with equal amplitudes but different phases, leading to the nonlinear relation $\gamma_{3D}=\mu\gamma_{1D}$ with $\mu=24$ (Fig. S3e). The effective energy results confirm this relation. The same procedure applied to the disclination-core states gives $\mu=2.76$ (Fig. S3f), with the effective energy results exhibiting similarly good agreement. However, under strong nonlinearity, noticeable deviations between the 1DSL and the 3D lattice emerge for both disclination-line and -core states. These deviations originate from state beating induced by spatial overlap and inter-state competition in the 3D lattice, which are not fully represented in the reduced 1DSL. Overall, these results establish a consistent nonlinear correspondence between higher-dimensional lattices and the 1DSL, along with the associated nonlinear energy shift behavior.

**Fig. S3. Nonlinear-strength and energy-shift correspondence between 2D/3D lattices and the corresponding 1DSLs.** (a) Nonlinear energy shift under corner-state excitation in the 2D disclination lattice with $\mu=1$. (b) Nonlinear energy shift under disclination-center-state excitation in the 2D disclination lattice with $\mu=2$. (c) Nonlinear energy shift under corner-state excitation in the 3D disclination lattice with $\mu=1$. (d) Nonlinear energy shift under disclination-center-state excitation in the 3D disclination lattice with $\mu=2$. (e) Nonlinear energy shift under disclination-line-state excitation in the 3D disclination lattice with $\mu=24$. (f) Nonlinear energy shift under disclination-core-state excitation in the 3D disclination lattice with $\mu=2.76$.

**S5: Failure of anchor-site mapping for the 2D disclination state**

Here, to demonstrate the failure of anchor-site mapping in the 2D disclination state, we compare the corresponding linear dynamical results obtained from anchor-site mapping and anchor-mode mapping in the 2D disclination lattice. In the anchor-site mapping scheme, a single disclination core site is selected as the anchor site to construct the 1DSL (Figs. S4a and S4b). In contrast, the anchor-mode mapping employs a two-site out-of-phase disclination state to generate the corresponding 1DSL. The results show that the 1DSL derived from anchor-site mapping fails to faithfully reproduce the dynamical evolution of the 2D disclination state. By comparison, the 1DSL obtained from anchor-mode mapping exhibits excellent agreement with the dynamical evolution of the 2D disclination state (Fig. S4c). This difference arises because the disclination-center state is intrinsically distributed over two neighboring sites with comparable amplitudes and opposite phases. Consequently, a single-site anchor cannot fully represent the underlying state structure, whereas the anchor-mode mapping preserves the essential spatial and phase characteristics of the excitation. These observations suggest that, in the presence of a multi-site disclination state, anchor-mode mapping provides a more physically consistent framework for constructing the corresponding 1DSL.

**Fig. S4. Linear dynamics of a 2D disclination state and the corresponding 1DSLs under different Lanczos mapping schemes.** (a) Schematic of a single anchor-site mapping configuration in the 2D disclination lattice. (b) Effective on-site potentials $\beta_{n}$ and nearest-neighbor couplings $\kappa_{n}$ of the corresponding 1DSL obtained via anchor-site mapping. (c) Comparison of the linear dynamical evolution of the 2D disclination-center state and the corresponding 1DSLs obtained from anchor-site mapping and anchor-mode mapping.

**S6: Nonlinear self-localization in the topologically trivial disclination lattice**

In the main text, we primarily investigate the nonlinear dynamical evolution of localized states in topologically nontrivial higher-dimensional disclination lattices and the associated 1DSLs. In the topologically trivial phase, although topologically protected localized states are absent, strong nonlinearity can still induce the formation of nonlinear localized states, such as solitons, leading to self-trapping. In this case, the Lanczos mapping remains capable of capturing this nonlinear self-localization process and reproducing the corresponding dynamical evolution in the 1DSL.

Specifically, we consider the nonlinear dynamics initiated by exciting a single disclination-core site in a 2D disclination lattice (Fig. S5a), which serves as the anchor site for the corresponding 1DSL (Fig. S5c1). In the linear regime, no localized disclination state is supported, and the excitation exhibits significant oscillatory behavior (Figs. S5b1 and S5c2). Under strong nonlinear excitation with $\gamma_{2D}=\mu\gamma_{1D}$, where $\mu=1$ and $\gamma_{1D}=10$, the excitation undergoes self-induced localization. Moreover, the nonlinear dynamical evolution of the original 2D lattice remains in excellent agreement with that of the associated 1DSL (Figs. S5b2 and S5c2). These results demonstrate that the proposed Lanczos mapping remains valid even in the topologically trivial phase and can accurately describe nonlinear self-trapping dynamics.

**Fig. S5. Nonlinear dynamical evolution in the topologically trivial 2D disclination lattice and its associated 1DSL.** (a) Schematic of the excitation configuration, where a single core site serves as the anchor site for the associated 1DSL. (b1, b2) Output intensity distributions of the 1DSL in the linear and strongly nonlinear regimes, respectively. (c1) Effective nearest-neighbor couplings $\kappa_{n}$ and on-site potentials $\beta_{n}$ of the associated 1DSL obtained via anchor-site mapping. (c2) Comparison of the dynamical evolution between the 2D lattice and the associated 1DSL in the linear and strongly nonlinear regimes.

**Reference**

[1]	C. Lanczos, "An iteration method for the solution of the eigenvalue problem of linear differential and integral operators," *Journal of Research of the National Bureau of Standards,* vol. 45, pp. 255–282, 1950.

[2]	C. C. Paige, "Accuracy and effectiveness of the Lanczos algorithm for the symmetric eigenproblem," *Linear Algebra and its Applications,* vol. 34, pp. 235-258, 1980.

[3]	N. S. Witte, "The exact realisation of the Lanczos method for a quantum many-body system," *Physics Letters A,* vol. 254, pp. 18-23, 1999.

[4]	G. Grosso, L. Martinelli and G. Pastori Parravicini, "Lanczos-type algorithm for excited states of very-large-scale quantum systems," *Physical Review B,* vol. 51, pp. 13033-13038, 1995.

[5]	B. N. Partlett, "*The symmetric eigenvalue problem*," Prentice-Hall, 1980.

[6]	G. H. Golub and C. F. Van Loan, "*Matrix computations*," JHU press, 2013.

[7]	A. Moroz, "Haydock’s recursive solution of self-adjoint problems. Discrete spectrum," *Annals of Physics,* vol. 351, pp. 960-974, 2014.

[8]	S. Yu, X. Piao, J. Hong and N. Park, "Interdimensional optical isospectrality inspired by graph networks," *Optica,* vol. 3, pp. 836-839, 2016.
